# Mg-Zn-Ca Alloys for Hemostasis Clips for Vessel Ligation: In Vitro and In Vivo Studies of Their Degradation and Response

**DOI:** 10.3390/ma13133039

**Published:** 2020-07-07

**Authors:** Yen-Hao Chang, Chun Chieh Tseng, Chih-Yeh Chao, Chung-Hwan Chen, Sung-Yen Lin, Je-Kang Du

**Affiliations:** 1 School of Dentistry, College of Dental Medicine, Kaohsiung Medical University, Kaohsiung 80708, Taiwan; edward590198@gmail.com; 2 Combination Medical Device Technology Division, Medical Devices and Opto-Electronics Equipment Department, Metal Industries Research & Development Centre, Lujhu Township, Kaohsiung 82151, Taiwan; cctseng0915@gmail.com; 3 Department of Mechanical Engineering, National Pingtung University of Science and Technology, Pingtung 91201, Taiwan; cychao@mail.npust.edu.tw; 4 Orthopedic Research Center, Kaohsiung Medical University, Kaohsiung 80708, Taiwan; hwan@kmu.edu.tw (C.-H.C.); tony8501031@gmail.com (S.-Y.L.); 5 Department of Orthopedics, College of Medicine, Kaohsiung Medical University, Kaohsiung 80708, Taiwan; 6 Division of Adult Reconstruction Surgery, Department of Orthopedics, Kaohsiung Medical University Hospital, Kaohsiung Medical University, Kaohsiung 80708, Taiwan; 7 Department of Orthopedics, Kaohsiung Municipal Ta-Tung Hospital, Kaohsiung Medical University, Kaohsiung 80145, Taiwan; 8 Regeneration Medicine and Cell Therapy Research Center, Kaohsiung Medical University, Kaohsiung 80708, Taiwan; 9 Department of Dentistry, Kaohsiung Medical University Hospital, Kaohsiung 80708, Taiwan

**Keywords:** Mg-Zn-Ca alloy, hemostasis clips, chitosan, L-glutamic acid, dip coating

## Abstract

To control the degradation rate of magnesium (Mg) alloys, chitosan (CHI) and L-glutamic acid (LGA) were used as coatings on Mg-Zn-Ca alloys via dip coating. In this study, either two or seven CHI/LGA layers were applied as a coating on Mg-2.8Zn-0.8Ca alloy (ZX31) and Mg-2.8Zn-0.8Ca hemostasis clips (ZX31 clips). The morphologies, compositions, and surface roughness of the specimens were characterized via scanning electron microscopy, Fourier transform infrared spectroscopy, and surface measurement devices. The degradation rates and behavior of the specimens were evaluated by immersing them in simulated body fluids and by applying these ZX31 clips on rabbits’ uterine tubes for five weeks. The specimen with seven layers (ZX31(CHI/LGA)_7_) exhibited improved corrosion behavior when compared with ZX31 or ZX31(CHI/LGA)_2_, with a reduced degradation rate of the Mg alloy in a simulated body environment. In vivo experiments showed that ZX31 clips exhibited good biocompatibilities in each group but could not maintain the clamping function for five weeks. The weight loss of ZX31(CHI/LGA)_7_ was significantly lower than that of the other groups. Consequently, it was verified that CHI can be used as a protective layer on a magnesium alloy surface via in vitro and in vivo experiments.

## 1. Introduction

Laparoscopic surgery (LS) has become a common surgical procedure because of its advantage in reducing the probability of injury, while causing little or no wound complications; consequently, this results in short hospital stays after the surgery [1,2,3]. During abdominal LS operations, hemostasis clips are one of the most common devices used for vessel ligation, replacing sutures. In commercial hemostasis clips, the most commonly used materials are titanium (Ti) or titanium alloys (Ti alloys) because of their biocompatibility, high strength, and good corrosion resistance [1,2]. However, their use may cause artefacts during magnetic resonance imaging (MRI) and in computed tomography (CT) scans because of the high X-ray absorption coefficient of Ti [1,2,3]. Case reports have also shown that clip migration (CM) can occur in some patients after surgery [4,5,6]. In addition to patient suffering and the requirement of additional surgery, CM is likely to cause other complications [6]. To overcome these problems, bio-absorbable polymer clips have recently been developed. However, the application of polymer clips has been limited due to their low strength [1]. 

Magnesium (Mg) and magnesium alloys (Mg alloys) have become the most prominent metallic biomaterials in recent years due to their biodegradability and good biocompatibility. Mg alloys can be absorbed by the body and eliminated via urine due to their chemical reaction with H_2_O. Furthermore, Mg and Mg alloys have many attractive physical characteristics, such as their density (1.74–2.0 g/cm^3^) and Young’s modulus (approximately 41–45 GPa) [7]. However, Mg alloys corrode too quickly when they are exposed to body fluids. The human body cannot absorb the entire amount of produced hydrogen, thus gas pockets are generated and bubbles are accumulated [8]. Additionally, uneven corrosion could result in corrosion-assisted cracking and loss of strength (risk of implant failure), as well as locally high pH values [9]. Among these, locally high pH values cause surrounding cells to undergo osmotic shock, resulting in tissue inflammation [9]. This limits the clinical applications of Mg alloys as medical implant devices. 

Numerous approaches have been proposed in the literature to improve corrosion resistance, such as alloying, cryogenic machining, and equal channel angular pressing (ECAP) [10,11]. In addition, surface modification of Mg alloys is also an effective approach for improving the corrosion resistance, such as micro-arc oxidation (MAO) coating, chemical conversion coating, electrophoretic deposition coating, and atomic layer deposition [12,13,14,15,16,17]. Furthermore, various polymers have been commonly used to coat the surfaces of Mg alloys to provide a protective layer and lower the corrosion rate (such as by dip coating). Among all the surface modification methods previously studied, the layer-by-layer (LbL) self-assembly technique is a simple and versatile method for incorporating polyelectrolytes, which is achieved by leveraging the electrostatic attractive forces between oppositely charged particles [12,18].

Recently, hydrogels made of natural polymers, such as alginate, collagen, chitosan (CHI), gelatin, and L-glutamic acid (LGA), have been used in numerous fields, especially as biomaterials [19,20,21]. Among these polymers, CHI and LGA have many reported applications in different fields, such as tissue engineering, regenerative medicine, and drug delivery [22,23,24,25,26,27,28]. Thus, CHI can be regarded as a favorable material for coating onto magnesium alloys due to its biocompatibility, low toxicity, immune-stimulatory activity, and biodegradability [24,29]. In addition, CHI possesses adhesive properties and superior film-forming ability [30]. 

Liangjian et al. [31] used CHI as a coating on Mg-based composites. The results demonstrated that CHI coating creates an effective corrosion-resistant layer that can decrease H_2_ release. Additionally, the results indicated that the CHI coating did not exhibit cytotoxicity towards L-929 cells. However, the protective power of CHI on the surface is still slightly insufficient, and the corrosion resistance of magnesium alloys needs to be strengthened. In this context, it may be possible to control the degradation rate of Mg alloys by employing multiple CHI protective layers. 

CHI becomes positively charged when CHI is dissolved in a solution with a pH value lower than its pKa. In order to create attractive forces between oppositely charged particles, LGA can be added, as it becomes negatively charged when the pH of the solution is higher than its pKa. Furthermore, the amino group of LGA loses an extra proton, meaning LGA becomes doubly negative (-OOC-CH(NH_2_)-(CH_2_)_2_-COO-) at pH values higher than 9.47. Thus, one of the negative charges of the LGA can adsorb the positively charged CHI and the other negative charge can adsorb the next positively charged CHI. In line with the above facts, polyelectrolyte multilayers (PEMs) can be fabricated by leveraging the electrostatic forces between CHI and LGA.

The aim of the present study is to fabricate a biocompatible Mg clip and assess the feasibility of realizing a multilayer coating using CHI as a polycation and LGA as a polyanion. The degradation characteristics of the fabricated clips were observed via in vitro and in vivo experiments.

## 2. Materials and Methods 

### 2.1. Materials and Specimen Preparation

**Figure 1:**
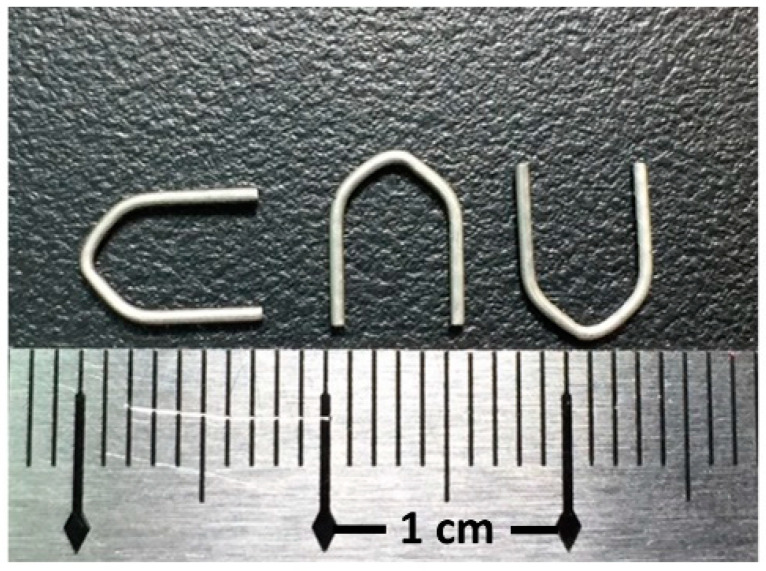
Hemostatic Mg clips.

Mg-2.8Zn-0.8Ca alloy (ZX31) was produced from highly pure Mg (99.9%), Zn (99.9%), and Ca (99.8%) using an electric resistance heating furnace with a mild steel crucible under argon atmosphere. ZX31 hot-extruded alloy bars (diameter, 10 mm) were obtained at 350 °C, with a reduction ratio of 10:8 via continuous extrusion (speed, 15 cm/min) of the ingot (diameter, 90 mm). The as-extruded ZX31 alloy was cut into 3-mm-thick disks, which were used as specimens for the experiments. The chemical composition of the alloy is shown in Table 1. A rectangular line of 1 mm × 0.8 mm was produced using the as-extruded ZX31 by further extrusion with the same parameters. Finally, the hemostasis clips were made using a self-made stamping machine. These clips were manufactured by the Metal Industries Research and Development Center (MIRDC, Kaohsiung, Taiwan) and are shown in Figure 1.

### 2.2. Preparation of the Coating 

**Figure 2:**
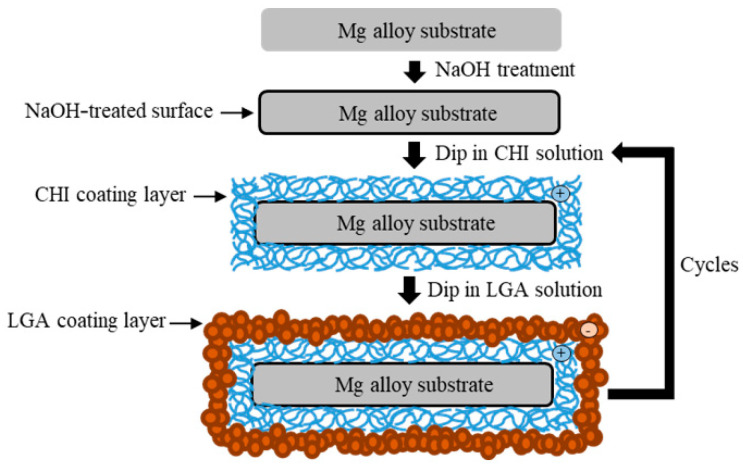
Schematic illustration of the chitosan (CHI) and L-glutamic acid (LGA) coating process.

The specimens were ground with SiC paper of up to #2000 and ultrasonically cleaned with ethanol. Before coating, the specimens were soaked in 5 M of NaOH solution for 10 min with stirring to obtain hydroxylate (MgOH_2_) on the surface. The specimens were again washed with ethanol and then dried with warm air. The surface of the alloy became negatively charged after etching [14]. CHI (medium molecular weight) and LGA were purchased from Sigma-Aldrich (St. Louis, MO, USA). A cationic CHI solution (0.6 wt.%) was mixed with a 0.5% acetic solution to obtain the coating mixture. The pH was then adjusted to 5.0 ± 0.2 using 5 M of NaOH solution. The pH of the anionic LGA solution (10 g/L) was adjusted to 9.7 using 5 M of NaOH solution. First, the pretreated specimens were dip-coated in the CHI solution for 10 min and dried at 110 °C for 1 h. Thereafter, the specimens were dip-coated in the LGA solution for 1 min and dried at 110 °C for 1 h. A schematic illustration of the coating process is shown in Figure 2. The number of layers of CHI coating was used to name the samples. In this study, samples with either two or seven layers of CHI were produced via the aforementioned dip coating process. The samples were denoted as substrate ZX31 (CHI/LGA)_2_ or ZX31 (CHI/LGA)_7_.

### 2.3. Surface Characterization

First, the coated surface of the specimen was gold-sputtered. Then, a scanning electron microscope (SEM) (S-3000N, Hitachi, Tokyo, Japan) with an energy-dispersive spectrometer (EDS) (E-MAX ENERGY, HORIBA, Kyoto, Japan) was used to examine the surface morphology and chemical composition of the samples at 15 kV. The surface characteristics and roughness of the coating layers were investigated using a 3-D surface measurement device (GBS, SmartWLI-Prime, Ilmenau, Germany). The roughness was measured using the surface roughness parameter Ra, which is the arithmetic average of the surface height values. Fourier transform infrared spectroscopy (FTIR) (MER Spectrum 100 FTIR, PerkinElmer, Waltham, MA, USA) was performed to analyze the chemistry of the CHI and LGA that were coated onto the ZX31 samples. Pellets of CHI and LGA were formed by compressing the samples in a stainless steel mold at high pressure. The spectra were calculated from 600 to 4000 cm^−1^, with a resolution of 4 cm^−1^. 

### 2.4. In Vitro Degradation Tests

The prepared samples were immersed in simulated body fluid (SBF) [32] at 37 °C for 30 days. In order to maintain a relatively stable immersion environment in vitro, the SBF solution was renewed every 24 h. Three samples were tested for each set of conditions. The hydrogen evolution method [28] was used to calculate the volume of hydrogen released. Using this method, hydrogen gas was collected and the amount of hydrogen released was calculated via the measured height difference of the liquid levels. The weight loss of the samples was used to calculate the corrosion rate (mm/year) through the following equation [33]:

Corrosion rate = (K × W)/(A × T× D)(1)

where K is a coefficient with a value of 8.76 × 10^4^, W is the weight loss (g), A is the area of the sample exposed to solution (cm^2^), T is the exposure time (h), and D is the density of the material (g/cm^3^).

### 2.5. Animal Surgery Study

Animal experiments were conducted in compliance with animal ethics regulations and were authorized by the Institutional Animal Care and Use Committee (No. 106170). Three adult female New Zealand rabbits weighing 3.0–5.0 kg each were used in the study to simulate the implantation of Mg clips during human surgery. We selected the rabbit’s uterine tube to simulate the implantation of the hemostatic clips because of its appropriate size. The animals were administered general anesthesia before the surgery, and the region to be operated on was scrubbed with a 25 g/L tincture of iodine and 70% ethanol. Afterwards, we randomly selected a rabbit and named it rabbit-1. Continuous hemostatic clips were applied on the left and right uterine tubes of rabbit-1. Three uncoated ZX31 clips and three ZX31(CHI/LGA)_2_ clips were attached to the left and right uterine tubes, respectively, of this rabbit. The uterine tubes were surgically put back into the animal and the wound was sutured. Then, we randomly selected a second rabbit (rabbit-2) and applied three ZX31 and three ZX31(CHI/LGA)_7_ clips to its left and right uterine tubes, respectively.

Finally, the left and right uterine tubes of the third rabbit were clipped with three ZX31(CHI/LGA)_7_ and three ZX31(CHI/LGA)_2_ Mg clips, respectively. The three rabbits were euthanized five weeks after surgery.

### 2.6. Histological and Radiographic Evaluations

Radiographs were obtained to observe the degradation process at two and three weeks after surgery. After the removal of the Mg clips, the soft tissue of the oviduct samples was dehydrated. The tissues were then embedded in paraffin and a histological evaluation was performed on hematoxylin-and-eosin-stained sections. The weight loss of each of the Mg clips was also measured using a precision balance (AS 220/C/1, RODWAG, Radom, Poland); this measurement was performed before and after the surgery.

### 2.7. Statistical Analysis 

One-way analysis of variance (ANOVA) testing was used to determine the level of significance, and multiple comparisons were performed using Scheffe’s method. Statistical differences were considered to be significant at *p* < 0.05.

## 3. Results and Discussion

### 3.1. Surface Morphology

**Figure 3:**
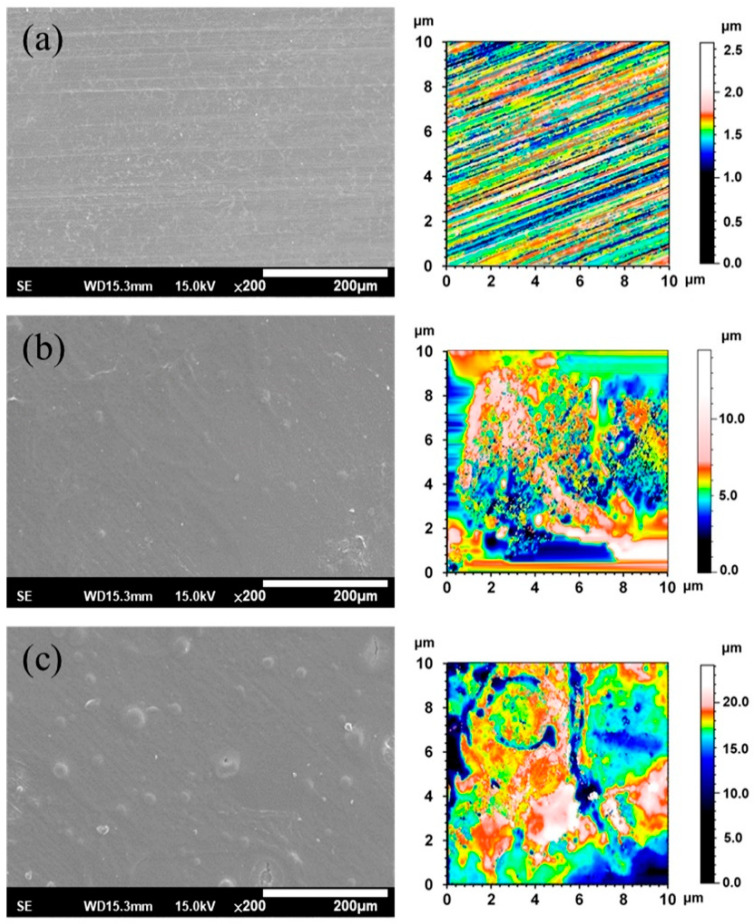
SEM and 2-D texture height maps of (**a**) a specimen ground with #2000 SiC paper; (**b**) ZX31(CHI/LGA)_2_ specimen; (**c**) ZX31(CHI/LGA)_7_ specimen.

Figure 3 shows the SEM morphologies and 2-D texture height maps of the ZX31, ZX31(CHI/LGA)_2_, and ZX31(CHI/LGA)_7_ specimens. The uncoated specimen had visible scratches, which were attributed to the grinding process (Figure 3a), and the surface roughness (Ra) was 1.19 µm. The specimens coated with CHI are shown in Figure 3b,c. In contrast, there were no scratches on the CHI-coated Mg specimens (Figure 3b), indicating that the films fully covered the substrates. The ZX31(CHI/LGA)_2_ and ZX31(CHI/LGA)_7_ specimens exhibited some CHI agglomerations on the substrate, and the agglomerations of the ZX31(CHI/LGA)_7_ specimen were larger than those of the ZX31(CHI/LGA)_2_ specimen. Mainly because of the coating process, the surfaces of initially small agglomerations continued to have CHI deposited onto them. As the number of electrodeposition layers increased, the height of the surface increased. The surface roughness of the CHI-coated specimens increased from 1.72 µm (two layers) to 2.60 µm (seven layers). The surface roughness of the CHI-coated specimens was high due to the different deacetylation degrees of CHI molecules and the corrosion of the Mg substrate during film formation [30]. 

### 3.2. Coating Composition

**Figure 4:**
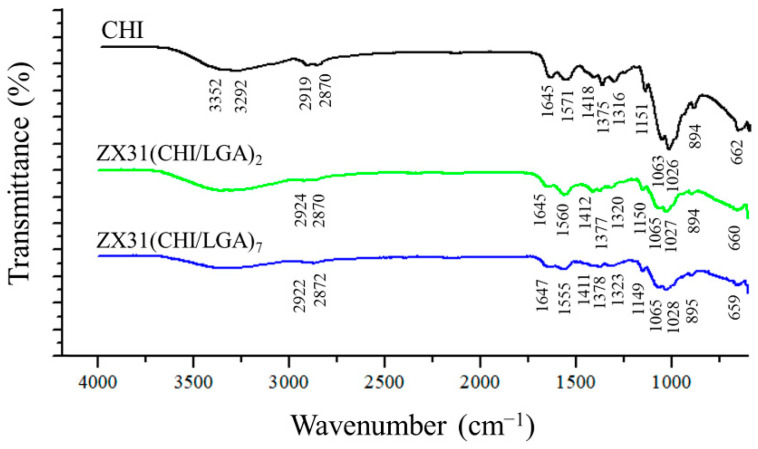
FTIR spectra of pure CHI, ZX31(CHI/LGA)_2_ and ZX31(CHI/LGA)_7_ specimens.

FTIR was employed to analyze the chemical composition of the surface of the coating (Figure 4). For comparison purposes, the FTIR spectrum of pure CHI was also measured. The spectra of the ZX31(CHI/LGA)_2_ and ZX31(CHI/LGA)_7_ specimens exhibited similar characteristic peaks to those of their parent polymers. In the spectrum of the ZX31(CHI/LGA)_7_ specimen, the characteristic saccharide peaks of CHI in the 961–1186 cm^−1^ region were observed. The C-O stretching absorption band at 1065 cm^−1^ and 1027 cm^−1^ and the C-O-C asymmetric stretching vibrations at 1149 cm^−1^ were also observed. The broad absorption line in the region between 3200 and 3600 cm^−1^ was attributed to -OH and -NH stretching, and the signals at approximately 1645, 1555, and 1378 cm^−1^ were attributed to the amide I, II, and III modes of the residual *N*-acetyl groups, respectively. In the coated specimens, high-frequency shifts of amide II were found, which were ascribed to the electrostatic interaction between the -COOH of LGA and the -NH_2_ of CHI [34]. This phenomenon was present with a more dramatic shift in the ZX31(CHI/LGA)_7_ specimen. Therefore, the results of the FTIR analysis clearly indicated the successful fabrication of CHI and LGA multilayers. Moreover, the spectra of the specimens also varied with the number of layers of CHI coating.

### 3.3. In Vitro Corrosion Resistance

**Figure 5:**
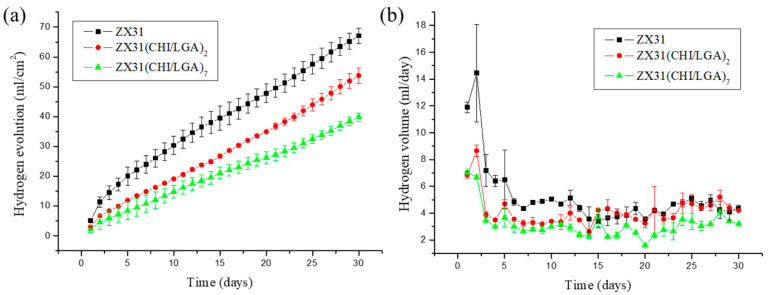
Volume of hydrogen for each of the specimens during a 30-day immersion test in SBF: (**a**) hydrogen evolution process of uncoated ZX31, ZX31(CHI/LGA)_2_, and ZX31(CHI/LGA)_7_ specimens immersed in an SBF solution at 37 ± 0.2 °C for 30 days; (**b**) H_2_ released per day.

The degradation rates of ZX31 specimens with or without a chitosan coating were measured in simulated body fluid (SBF) at 37 ± 0.2 °C for 30 days. The released hydrogen (H_2_) volumes of the different specimens were measured to analyze the effect of the degradation rate of the coating and understand its corrosion behavior. The degradation rates of the different specimens are shown in Figure 5a. It can be seen that uncoated ZX31 exhibited a high degradation rate during the 30 days of immersion. Moreover, it can be seen that the degradation rates of the CHI-coated specimens were markedly low during this period. The average degradation rates of the ZX31, ZX31(CHI/LGA)_2_, and ZX31(CHI/LGA)_7_ specimens were 5.12, 4.11, and 3.03 mm/y, respectively. The specimen with seven layers of CHI coating exhibited a lower degradation rate when compared with that for the specimen with two layers. The improved corrosion resistance of the ZX31(CHI/LGA)_2_ and ZX31(CHI/LGA)_7_ specimens when compared with the ZX31 specimen can be attributed to the use of polyelectrolyte multilayers (PEMs). Moreover, an increase in the number of layers of CHI coating can delay the corrosion of the Mg substrate in corrosive environments. 

Figure 5b shows that the uncoated ZX31 released a higher amount of H_2_ per day than the coated specimens during the first immersion stage of seven days. It can be noted that the amount of H_2_ released per day was similar in the ZX31 and ZX31(CHI/LGA)_2_ specimens, as shown in Figure 5b. After seven days of immersion, the volume of H_2_ released was low and stable in all the specimens. This implies that the anticorrosion effect of CHI plays a significant role only in the early stage. The surface of the ZX31(CHI/LGA)_7_ was completely covered by the CHI, which acts as a protective layer to prevent environmental corrosion. After CHI degrades, the coated specimens lose their protection and their area of exposure increases, which consequently increases their degradation rate. 

These in vitro results show that the degradation rate can be reduced by increasing the number of coating layers. Liangjian et al. [31] analyzed Mg-based composites that were coated with CHI and studied the in vitro degradation behavior in SBF. The results of their study showed that the CHI-coated specimens exhibited higher corrosion resistance than did the uncoated specimens. Moreover, the results of the in vitro cytotoxicity tests indicated that the CHI-coated specimens were safe for medical use. Bai et al. [35] fabricated Mg-Zn-Ca alloys via MAO to produce porous surfaces. These specimens were dip-coated in a CHI solution to fill in the holes of the MAO surface. They concluded that CHI coating is a promising strategy for slowing down the degradation of Mg alloys. Furthermore, Cui et al. [12] produced an AZ31 (composition in mass fraction, %: Al 2.5–3.5, Zn 0.6–1.4, Mn 0.2 and Balanced Mg) alloy with CHI and poly-LGA coatings via LbL methods. The coated specimens that they used exhibited high corrosion resistance, as well as antibacterial properties. All these studies demonstrate the various methods employed for fabricating CHI coatings, which were a key factor for controlling the corrosion resistance of Mg alloys. This can be attributed to the fact that CHI undergoes relatively slow biodegradation in SBF (or physiological environments).

### 3.4. EDS Analysis

**Figure 6:**
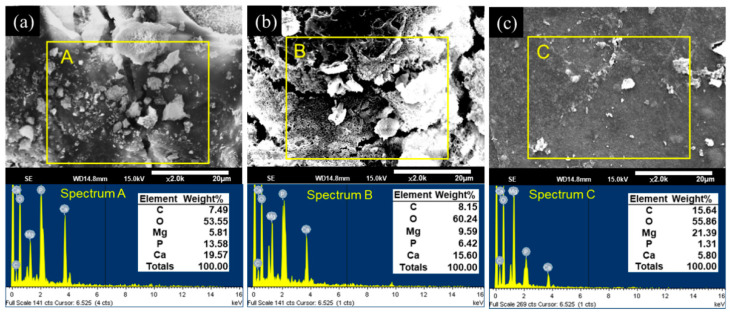
SEM observations and EDS point spectrum analysis of the corroded surfaces after 30 days of immersion for (**a**) ZX31; (**b**) ZX31(CHI/LGA)_2_ and (**c**) ZX31(CHI/LGA)_7_.

After the in vitro immersion test, the morphologies and elemental compositions of the degraded surfaces of the uncoated and CHI-coated specimens were studied using SEM and EDS. These results are shown in Figure 6. After 30 days of immersion, both the ZX31 and the ZX31(CHI/LGA)_2_ specimens exhibited different surface morphologies with corrosion products on the surface. The rugged surface morphology of the ZX31 and ZX31(CHI/LGA)_2_ specimens revealed that severe degradation had occurred.

In addition, particles with various shapes and different sizes could be also seen on the surface of the specimens. EDS analysis showed that these particles were mainly composed of Mg, O, C, Ca, and P. Pan et al. [36] proved that these particles were bone-like apatites. Furthermore, the amount of corrosion products on the surface of the ZX31(CHI/LGA)_7_ specimen after 30 days was lower than on the ZX31 and ZX31(CHI/LGA)_2_ specimens. This implies that the ZX31(CHI/LGA)_7_ specimen was more stable than the other two specimens. 

### 3.5. In Vivo Estimation

**Figure 7:**
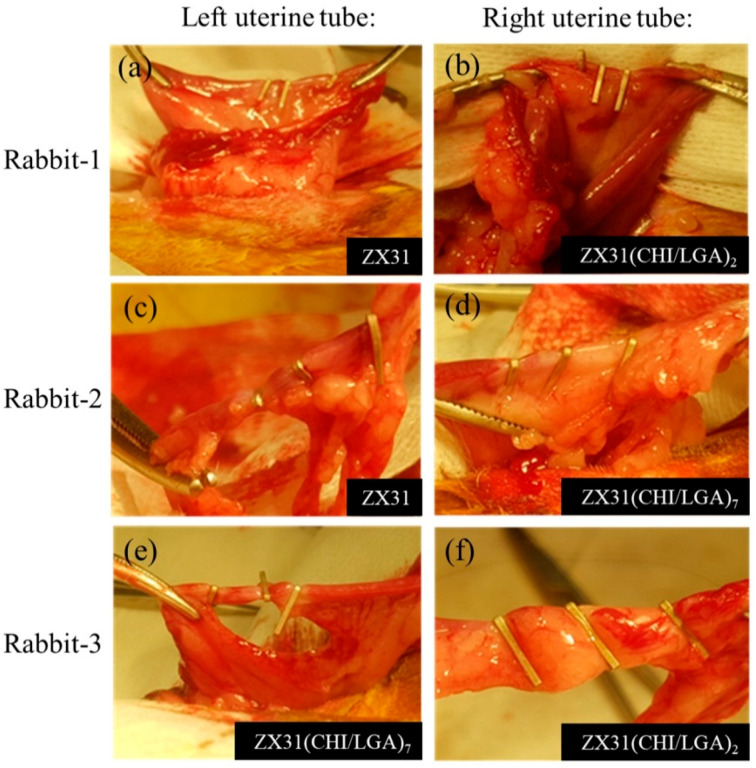
Placement of Mg clips on the uterine tubes of rabbits: (**a**,**b**) rabbit-1, for which three ZX31 and three ZX31(CHI/LGA)_2_ Mg clips were used; (**c**,**d**) rabbit-2, for which three ZX31 and three ZX31(CHI/LGA)_7_ Mg clips were used; (**e**,**f**) rabbit-3, for which three ZX31(CHI/LGA)_7_ and three ZX31(CHI/LGA)_2_ Mg clips were used.

Figure 7 shows the surgical procedures in which the Mg clips were used in the uterine tubes of each rabbit. Herein, Figure 7a,b displays the ZX31 and ZX31(CHI/LGA)_2_ Mg clips on the left and right uterine tubes of rabbit-1 after occlusion, respectively. Figure 7c,d displays the ZX31 and ZX31(CHI/LGA)_7_ Mg clips on the left and right uterine tubes of rabbit-2 after occlusion, respectively. Moreover, Figure 7e,f displays the ZX31(CHI/LGA)_7_ and ZX31(CHI/LGA)_2_ Mg clips on the left and right uterine tubes of rabbit-3 after occlusion, respectively. The results show that the clips can completely close the uterine tubes of rabbits. All three animals survived and there was no swelling, rupture, or dehiscence of the wounds throughout the observation period. The wounds were observed to heal two weeks after surgery, and a small amount of newly sprouted hair was seen in the abdominal area of the rabbits. These results suggest that Mg clips can safely close the uterine tubes of rabbits for at least two weeks. 

### 3.6. In Vivo X-ray Images

**Figure 8:**
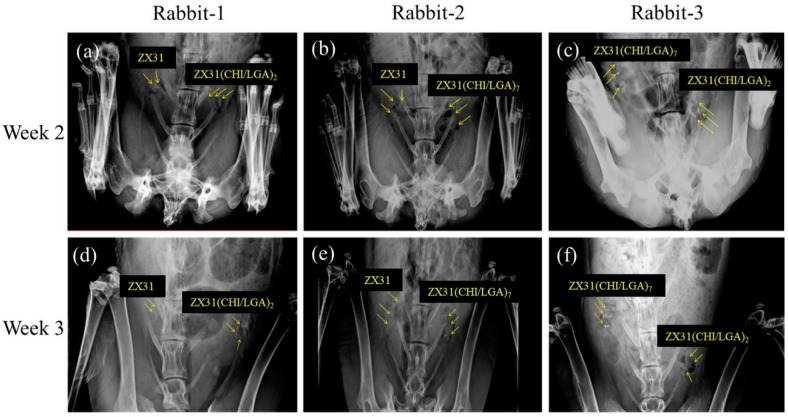
X-ray images of rabbits with Mg clips taken two and three weeks after surgery: (**a**) ZX31 and ZX31(CHI/LGA)_2_ Mg clips on the left and right uterine tubes of rabbit-1, respectively (second week); (**b**) ZX31 and ZX31(CHI/LGA)_7_ Mg clips on the left and right uterine tubes of rabbit-2, respectively (second week); (**c**) ZX31(CHI/LGA)_7_ and ZX31(CHI/LGA)_2_ Mg clips on the left and right uterine tubes of rabbit-3, respectively (second week); (**d**–**f**) the clips in the third week.

All rabbits survived the predefined implantation duration. Figure 8 shows X-ray images of the rabbits with Mg clips at two and three weeks after the surgery. Figure 8a–c displays the position and condition of the clips in the rabbits two weeks after surgery. The images show that the clips could maintain their original shape during this period. Figure 8d–f displays the position and condition of the clips in the rabbits three weeks after surgery. The images show that the clips could maintain their grip after occlusion of the uterine tube. They also show that no significant displacement or migration occurred during this period.

### 3.7. Autopsy and Histological Analysis

The rabbits were euthanized under painless conditions. At the time of autopsy, no clinical abnormalities were found in the peritoneal cavity for either the coated or uncoated Mg clips. There was no excess production of hydrogen gas, which is the main concern preventing the practical use of Mg-Zn-Ca instruments. The excellent biocompatibility and occlusion ability of the clips were confirmed in this first stage of our study. In the next stage, it will be necessary to monitor the inosculation of actual blood vessels of animals under different conditions and diseases. 

**Figure 9:**
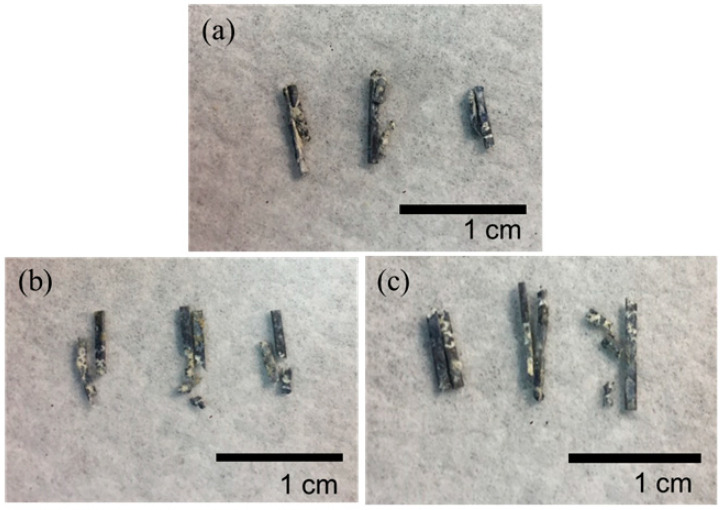
Mg clips taken from the rabbits five weeks after surgery: (**a**) ZX31 clips; (**b**) ZX31(CHI/LGA)_2_ clips; (**c**) ZX31(CHI/LGA)_7_ clips.

**Figure 10:**
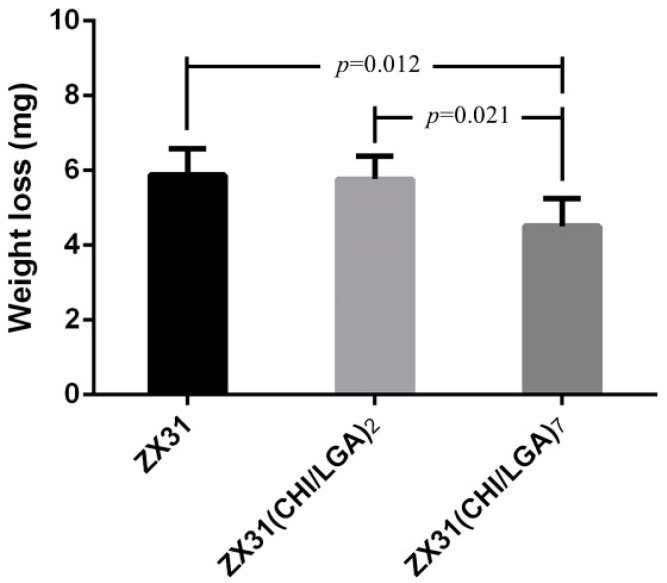
Weight loss of the Mg clips from each group five weeks after surgery (*p* values obtained via Scheffe’s method).

Figure 9 shows the remainder of the Mg clips of each group five weeks after the surgery. It can be seen that all the groups of Mg clips lost their original shape and function. Additionally, the weight loss values of the Mg clips are compared in Figure 10. The average weight loss of ZX31(CHI/LGA)_7_ clips was 4.51 ± 0.73 mg, which was significantly lower than for both ZX31 and ZX31(CHI/LGA)_2_ at 5.88 ± 0.69 mg and 5.76 ± 0.61 mg, respectively. This indicated that ZX31(CHI/LGA)_7_ could decrease the degradation rate of the Mg-Zn-Ca alloy in vitro, but not enough to retain function.

In this study, CHI coating was used to control the rate of degradation of the Mg clips. The in vivo experiments on animals produced better results when compared with the in vitro experiments. We speculate that this is because the CHI coating was present on the surface of the clips. Therefore, as the CHI surface layer was not absorbed because of its low degradation rate, it provided a protective effect for the Mg clips. It is noted that ZX31(CHI/LGA)_2_ did not present any in vitro corrosion resistance when compared with ZX31. The coating was breached because the Mg clip has many edges and corners, and the two layers of the CHI coating were, in theory, unable to effectively cover the corners and edges. Therefore, the corrosive solution seeped through the corners and edges and came into contact with the magnesium substrate beneath the coating. Consequently, the seven-layer CHI coating was able to provide the anticipated level of protection. However, the mechanical properties of the Mg clips also have to be considered. Hence, it is necessary to clarify whether micro-cracks appeared in the Mg clips after the clipping procedure, which could have accelerated their corrosion, ultimately resulting in their fracture. 

The main reason why the rate of degradation of the Mg clips must be controlled is to minimize the effects of the release of large amounts of hydrogen [37]. Negative effects due to the release of hydrogen were not observed during our experiments. This may be because the Mg clips were small and the hydrogen released by the clips was simply consumed by the animals’ metabolic reactions. Therefore, applications involving a large number of Mg clips must still be investigated in the future. A closed incisional wound takes a week to heal; thus, the clip device must be able to keep the wound closed during this time [2]. In Figure 8d–f, it is clearly shown that the various Mg clips were able to maintain closure of the wound for three weeks. 

**Figure 11:**
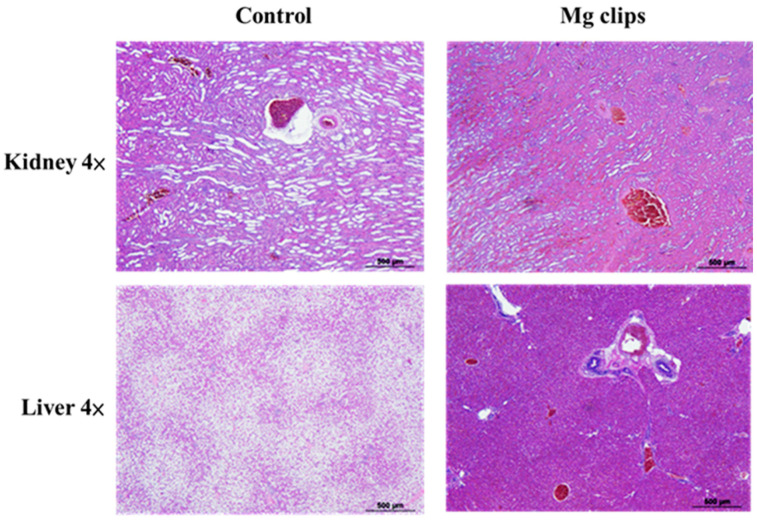
Histological photographs of hematoxylin-and-eosin-stained sections of tissues.

Figure 11 shows hematoxylin-and-eosin-stained, paraffin-embedded liver and kidney sections of the rabbits that were implanted with the coated Mg clips. No differences were observed between these rabbits and normal rabbits without Mg clip implantation in their liver and kidney sections. These results indicate that the Mg alloy with CHI coating showed good biocompatibility with animal organs (kidney, liver) [38]. The animals in the experiment exhibited good tolerance towards the products released due to the degradation of ZX31 and ZX31(CHI/LGA) alloy implants. Therefore, it is evident that the implanted clips are biocompatible in the early stage.

## 4. Conclusions

To achieve controllable degradation, protective and biocompatible composite layers of CHI and LGA were successfully electrodeposited onto an Mg-Zn-Ca surface. ZX31(CHI/LGA)_7_ clips exhibited the lowest degradation rate in both in vitro and in vivo experiments. These coating layers acted as an effective barrier for the Mg substrate against corrosive environments. The specimen with the higher number of CHI coating layers exhibited a lower proportion of Ca and P on its surface, which means that it had a lower degradation rate. In vivo evaluation of the occlusion properties of the fabricated Mg-Zn-Ca clips showed that they could completely close the uterine tubes of rabbits. However, this did not result in significant differences between ZX31 and the coated ZX31(CHI/LGA)_2_ or ZX31(CHI/LGA)_7_ specimens with regard to the function of the clips themselves. Both the uncoated and coated Mg clips exhibited excellent biocompatibility. Therefore, it can be concluded that the newly developed Mg-Zn-Ca clips are strong candidates for biodegradable soft-tissue fixation devices, such as hemostasis clips. 

## Figures and Tables

**Table 1:** Composition of the Mg-Zn-Ca alloy (wt.%).

Element	Mg	Zn	Ca	Al	Si
wt.%	Bal.	2.83	0.78	0.04	0.01
